# A Genomic Outlook on Bioremediation: The Case of Arsenic Removal

**DOI:** 10.3389/fmicb.2018.00820

**Published:** 2018-04-26

**Authors:** Frédéric Plewniak, Simona Crognale, Simona Rossetti, Philippe N. Bertin

**Affiliations:** ^1^Génétique Moléculaire, Génomique et Microbiologie, UMR7156 CNRS, Université de Strasbourg, Strasbourg, France; ^2^Istituto di Ricerca sulle Acque, Consiglio Nazionale delle Ricerche, Rome, Italy

**Keywords:** genomics, arsenic, bioremediation/phytoremediation, microorganism, ecosystem ecology

## Abstract

Microorganisms play a major role in biogeochemical cycles. As such they are attractive candidates for developing new or improving existing biotechnological applications, in order to deal with the accumulation and pollution of organic and inorganic compounds. Their ability to participate in bioremediation processes mainly depends on their capacity to metabolize toxic elements and catalyze reactions resulting in, for example, precipitation, biotransformation, dissolution, or sequestration. The contribution of genomics may be of prime importance to a thorough understanding of these metabolisms and the interactions of microorganisms with pollutants at the level of both single species and microbial communities. Such approaches should pave the way for the utilization of microorganisms to design new, efficient and environmentally sound remediation strategies, as exemplified by the case of arsenic contamination, which has been declared as a major risk for human health in various parts of the world.

## From Genes to Metagenomes

In over three billion years of evolution, microorganisms have colonized nearly all ecological niches, including the most extreme environments. Due to their multiple metabolic activities, they play a major part in biogeochemical cycles, affecting soil productivity or water quality (Madsen, 2011) and constitute an immense reservoir of genes with high potentials for biotechnology applications. For those reasons, microorganisms from the environment have aroused a strong interest since long before the microbial genomics era. A large number of enzymes and genes coding for biocatalyzers (cellulases, proteases, lipases/esterases, glycosidases, chitinases, xylanases, phosphatases) or for enzymes involved in vitamin and antibiotic biosynthesis have thus been isolated from environmental microorganisms (Colin et al., 2015; Jacques et al., 2017; Krüger et al., 2018). Many of these enzymes have been used for research, industrial or pharmaceutical applications (Madhavan et al., 2017) like, for instance, restriction enzymes and the Taq DNA polymerase that sparked a revolution in molecular biology techniques (Ishino and Ishino, 2014).

More than 20 years ago, thanks to the rise of molecular biology and the automation of DNA sequencing, microbiology embraced genomics, the ensemble of approaches which address the organization and activity of organisms within the scope of their full genome, acknowledging that no living system can be reduced to a single gene expressed at some time or another (Bertin et al., 2015). Since the very first genome sequence from a free-living organism, *Haemophilus influenzae* Rb (Fleischmann et al., 1995), the number of new microbial genome sequences published each year has grown exponentially to reach in 2014 a total of over 30,000 publicly available sequenced bacterial genomes (Land et al., 2015).

Yet, diversity data provided by molecular methods suggest that there remains in many ecosystems a vast majority of microorganisms belonging to taxa that have not been isolated in pure culture (Rashid and Stingl, 2015) and cultivation may be extremely difficult for a majority of them. Environmental genomic approaches could nonetheless provide access directly to the genome of uncultivated organisms like ‘*Candidatus* Desulforudis audaxviator,’ which practically represents the sole species present in a gold mine and can fix nitrogen using a cellular mechanism similar to that of Archaea (Chivian et al., 2008). Metagenomic analyses of nitrogen metabolism in anaerobic enriched cultures also led to the reconstruction of prokaryotic genomes such as *Kuenenia stuttgartiensis* (Strous et al., 2006), ‘*Candidatus* Nitrospira defluvii’ (Lücker et al., 2010) or the archaeon ‘*Candidatus* Methanoperedens nitroreducens’ (Haroon et al., 2013) involved in oceanic ammonium oxidation, nitrite oxidation in sewage treatment plant sludge and anaerobic oxidization of methane coupled to nitrate reduction, respectively. Similarly, the genome of an iron-oxidizer strain belonging to the *Ferrovum* genus was reconstructed from a mixed culture grown from samples collected in a mine water treatment plant (Ullrich et al., 2016).

Though molecular techniques associated with bioinformatic and genome-mining methods are invaluable tools to reveal the potential in genome data (Machado et al., 2017; Vallenet et al., 2017), cultivation remains an important challenge in microbiology, necessary for expanding our knowledge of microorganisms’ physiology and for bioremediation (Overmann et al., 2017). However, microorganisms from the environment may require essential nutrients or particular growth conditions, or may be extremely slow growers or obligate symbionts. Although tackling these issues generally demands strenuous efforts to design and test many isolation media, genome characterization may highlight metabolic characteristics of the targeted organism that could be leveraged to select and cultivate a given strain (Garza and Dutilh, 2015). This strategy allowed the isolation of the first nitrifying archaeon (Schleper et al., 2005) after an analysis of the Sargasso Sea metagenome (Venter et al., 2004) had detected on the same DNA fragment an Archaea-specific ribosomal gene and a gene coding for the ammonium monooxygenase, a key enzyme in nitrification. Subsequent physiological studies showed that the nitrification function was indeed expressed (Könneke et al., 2005). Another example is provided by the isolation of *Leptospirillum ferrodiazotrophum* (Tyson et al., 2005), which a previous metagenomic study had shown to be the only strain in an acid mine tailing to be able to fix nitrogen.

Beyond approaches centered on single organisms, the developments of genomics have rendered possible a global view of microbial communities that could help a better understanding of natural remediation processes and identifying candidate species for the design of bioremediation treatment plants. In this respect, high-throughput tools such as microarrays have allowed to address ecological questions related to the structure and function of microbial communities. Developed from the genomic data present in databases, such approaches may be helpful to study the diversity and dynamics of microbial populations using nucleic acids extraction and hybridization (Zhou et al., 2015). They were successfully used, for example, to examine the responses of microbial communities after the wreck of a drilling rig in the Gulf of Mexico had released about 5 million barrels of crude oil (Beazley et al., 2012). This study suggested that the microbial community of the rhizosphere in the affected coastal salt marsh could strongly contribute to hydrocarbon natural remediation. Recently, the combination of ribosomal 16S RNA gene high-throughput sequencing with DNA-based stable isotope probing in activated sludge samples incubated with Na_2_^13^CO_3_ uncovered the dynamics of ammonium-oxidizing microorganism abundance and the relative importance of archaeal and bacterial ammonium oxidation activities in a waste water treatment plant (Pan et al., 2018).

In recent years, the development of high-throughput sequencing and assembly software has allowed to determine the complete genome sequence of uncultivated microorganisms from direct sequencing of metagenomic libraries or environmental DNA from complex microbial communities. Despite a number of critical issues regarding sampling, assembly or annotation (Teeling and Glöckner, 2012; Thomas et al., 2012), more than 10,000 metagenome projects are now referenced in the Genomes Online Database (Mukherjee et al., 2016). This number is expected to increase dramatically with such massive projects as the Earth Microbiome Project whose goal is to produce a global Gene Atlas of microbial communities encompassing an estimated 500,000 genomes (Gilbert et al., 2010; Thompson et al., 2017).

**FIGURE 1:**
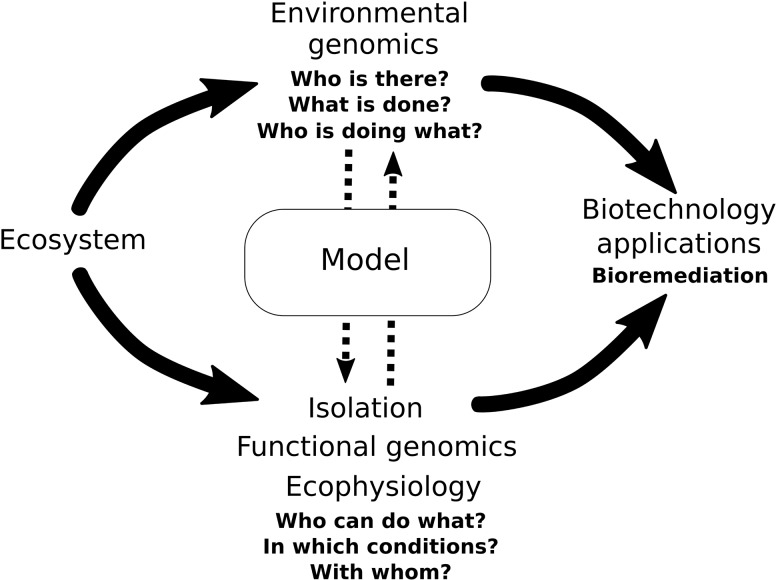
Multi-omic studies of microorganisms from the environment provide an integrated image or model of the microbial processes involved in the ecosystem’s functioning and that may be utilized for bioremediation or other biotechnology applications.

Environmental genomics now permits the study of the organisms in an ecosystem as a set of elements behaving within a complex network of interactions (**Figure 1**). For example, a genome-scale study of the complex symbiosis between the termite *Macrotermes natalensis*, its domesticated fungus and several gut bacterial communities demonstrated the cooperation between microorganisms in plant biomass conversion. The results showed that the insect provides the infrastructure allowing carbohydrate decomposition thanks to the functional complementarity between the fungus and the gut microbiota (Poulsen et al., 2014). More recently, the reconstruction of 2540 genomes using metagenomic data from 15 different sediment and groundwater environments allowed to highlight the key inter-organism interactions relevant to biogeochemical cycles in an aquifer in Colorado, United States (Anantharaman et al., 2016). Applying those approaches to various biotopes may thus provide valuable insights into the functioning of ecosystems, including polluted environments whose microbial communities could constitute prospective candidates for bioremediation.

## Arsenic Bioremediation and ‘Omics’ Approaches: a Case Study

Long-term exposure to arsenic represents a serious threat to human health worldwide (Nordstrom, 2002). Even though the occurrence of this element in drinking water constitutes the major source of exposure, recent studies on risks of arsenic accumulation in food revealed its presence in fish and crops cultivated with arsenic-contaminated waters (WHO, 2011; Jackson et al., 2012; Molin et al., 2015; Carlin et al., 2016). Numerous physico-chemical methods are commonly used for the treatment of arsenic-rich waters: coagulation/filtration, ion exchange, enhanced lime softening, adsorption and reverse osmosis (Ng et al., 2004; Nicomel et al., 2016). Over the last years, in a search for sustainable and cost-effective methods for water treatment, arsenic remediation turned to the potentialities of biological approaches. The use of rhizosphere microorganisms was recently investigated for their capacity to enhance phytoremediation of arsenic-contaminated environments (Ma et al., 2016). In particular, several arsenic-resistant microorganisms belonging to various genera, e.g., *Bacillus*, *Achromobacter*, *Brevundimonas*, *Microbacterium*, *Ochrobactrum*, *Pseudomonas*, *Comamonas*, *Stenotrophomonas*, *Ensifer* were reported to decrease toxic effects of arsenic and enhance plant growth by acting on arsenic mobilization and accumulation in plants (Cavalca et al., 2010; Ghosh et al., 2011; Wang et al., 2011; Yang et al., 2012; Pandey et al., 2013; Mallick et al., 2014, 2018; Mesa et al., 2017). The ability of fungi to resist, solubilize, transform or uptake metal species could also be used in mycoremediation of arsenic-contaminated soil (Singh et al., 2015; Srivastava et al., 2011). The production of volatile trimethylarsine by reductive methylation from inorganic and methylated arsenic compounds was reported in several fungal strains, e.g., *Aspergillus glaucum*, *Candida humicola*, *Scopulariopsis brevicaulis*, *Gliocladium roseum*, *Penicillium gladioli,* and *Fusarium* spp. (Cullen and Reimer, 1989; Lin, 2008). Bioaugmentation could thus represent a strategy to enhance the efficiency of As removal from waters and soils by the addition of specialized bacteria or fungi, either natural or genetically engineered, able to directly remove As by volatilization (Edvantoro et al., 2004; Chen P. et al., 2017) or indirectly through the formation of biogenic Fe-Mn oxides (Bai et al., 2016). However, despite an increasing interest for mycoremediation and rhizoremediation of arsenic contamination, still very little is known about their scalability.

To date, the bioremediation of arsenic-rich environments is mainly based on the use of microorganisms able to resist or metabolize arsenic through oxidoreduction reactions (Huang, 2014). Over the last decades the ecology of arsenic has been widely studied and several arsenic-metabolizing microorganisms isolated from various ecosystems have been characterized at the genomic level (Oremland and Stolz, 2003; Páez-Espino et al., 2009; Andres and Bertin, 2016). *Herminiimonas arsenicoxydans* was the first arsenic-metabolizing bacterium to be described. This β-proteobacterium isolated from an industrial wastewater treatment plant in Germany was shown to resist to high levels of arsenic and to oxidize arsenite, As(III), into arsenate, As(V) (Muller et al., 2007). Functional genomics demonstrated that this arsenic response is biphasic: *H. arsenicoxydans* activates the resistance response based in part on the induction of efflux mechanisms before inducing the detoxification processes leading to As(III) oxidation (Cleiss-Arnold et al., 2010; Koechler et al., 2010). Additionally, electron microscopy revealed that the strain is able to sequester arsenic within an exopolysaccharide (EPS) matrix (Muller et al., 2007). *Thiomonas* sp. 3As isolated from an abandoned mine in France was also shown to produce large amounts of EPS in the presence of arsenite, making it a good candidate for the development of bioremediation strategies relying on biofilm-based bioreactors (Arsène-Ploetze et al., 2010). A strain belonging to the *Rhizobium* genus isolated from an Australian gold mine was shown to carry arsenic resistance and detoxification genes on a large plasmid, which could provide an interesting genetic tool to transfer arsenic detoxification capacity into closely related plant-associated bacteria with the perspective of phytoremediation (Andres et al., 2013). More recently, the genome of two arsenite-oxidizing strains hyper-tolerant to arsenite was fully described: *Halomonas* A3H3 isolated from multicontaminated sediments in Mediterranean Sea (Koechler et al., 2013), and *Pseudomonas xanthomarina* S11 isolated from an arsenic-contaminated former gold mine in France (Koechler et al., 2015). Overall, the identification and the exploitation of microbial metabolic potentialities for arsenic-contaminated water treatment are considered an emerging challenge as mirrored by an increasing number of recent studies (Crognale et al., 2017). Among the available bacterial-driven processes, bioprecipitation, biosynthesis of adsorbent materials, biosorption and biovolatilization, involving several microorganisms (**Table 1**), are the most interestingly described for bioremediation of arsenic-contaminated waters (Fazi et al., 2016).

**Table 1:** Microorganisms used in As-removal processes from waters.

Microorganism	Process	Reference
*Ralstonia eutropha*	Bio-adsorption	Mondal et al., 2008
*Rhodopseuodomonas palustris*	Bio-volatilization	Liu et al., 2011
*Sphingomonas desiccabilis*	Bio-volatilization	Liu et al., 2011
*Bacillus idriensis*	Bio-volatilization	Liu et al., 2011
*Cyanobacteria*	Bio-volatilization	Yin et al., 2011
*Klebsiella oxytoca*	Bio-synthesis of adsorbent materials	Casentini et al., 2015
Mixed microbial community	Bio-precipitation	Omoregie et al., 2013
*Gallionella ferruginea* and *Leptothrix ochracea*	Microbial Fe-oxidation coupled to As removal	Katsoyiannis and Zouboulis, 2004
*Desulfotomaculum auripigmentum*	As and Fe–As sulfide precipitation driven by sulfate reducers	Newman et al., 1997
Mixed Sulfate Reducing Bacteria	As removal driven by sulfate reduction processes	Teclu et al., 2008; Serrano and Leiva, 2017
Mixed microbial community	Microbial Fe- and Mn-oxidation coupled to As removal	Thapa Chhetri et al., 2014
Mixed microbial community	As removal via co-oxidation with Fe and sorption or co-precipitation with Fe(III) (oxyhydr)oxides	Nitzsche et al., 2015
Mixed microbial community	Microbial Fe- and Mn-oxidation coupled to AsIII removal	Yang et al., 2014
Mixed microbial community	As(III) microbial oxidation coupled to FeII oxidation	Kamei-Ishikawa et al., 2017
*Aliihoeflea* sp. *2WW*	As(III) microbial oxidation	Corsini et al., 2014
*Thiomonas arsenivorans*	As(III) microbial oxidation	Wan et al., 2010; Dastidar and Wang, 2012
*Rhodococcus equi*	As(III) microbial oxidation	Bag et al., 2010
CAsO1 bacterial consortium	As(III) microbial oxidation	Battaglia-Brunet et al., 2002; Michel et al., 2007
*Ensifer adhaerens*	As(III) microbial oxidation	Ito et al., 2012
Mixed microbial community	As(III) microbial oxidation	Gude et al., 2018
Mixed microbial community	As(III) microbial oxidation	Li et al., 2016
Mixed microbial community	As(III) microbial oxidation	Sun et al., 2011
Mixed microbial community	Anoxic As(III) microbial oxidation coupled with chemolithotrophic denitrification	Sun et al., 2010

In recent years, several environmental genomic studies of arsenic-contaminated ecosystems have been conducted (Huang et al., 2016) and the molecular mechanisms involved have been recently reviewed in detail (Andres and Bertin, 2016). A metagenomic study of an acid mine drainage in France yielded nearly complete reconstructions of seven microbial genomes, providing a better understanding of the arsenic metabolism and natural attenuation which significantly reduce arsenic concentration along the creek, thanks to arsenite oxidation followed by co-precipitation with iron and sulfur. This analysis led to the identification of the corresponding genes, in particular *aio* coding for arsenite oxidase in *Thiomonas* sp. and *rus* coding for rusticyanin in *Acidithiobacillus* sp. (Bertin et al., 2011). A comparative metagenomic study of sediments in two harbors on the Mediterranean French coast, focusing on sequence markers specific for sulfur-metabolizing bacteria uncovered a correspondence between biotic sulfate reduction and the abiotic production of highly soluble thioarsenical compounds. In combination with arsenate reduction these processes, which favor arsenic dispersion in the water column, could explain the higher mobility of arsenic observed on the most contaminated site (Plewniak et al., 2013). Recently, the assembly of 27 Micrarcheota and 12 Parvarchaeota new genomes from 12 acid mine drainage and hot spring metagenomes was reported in a study targeting Archaeal Richmond Mine Acidophilic Nanoorganisms. The analysis of these almost complete genomes suggests a possible contribution of these organisms to carbon and nitrogen cycling by organic matter degradation, as well as to iron oxidation (Chen L.-X. et al., 2017). Those studies suggest that arsenic bioremediation strategies could be based upon microbial communities with iron, sulfur, and arsenic metabolism capacities and highlight the importance of metabolisms other than those of metals in arsenic removal. In this respect, mixed microbial communities were tested for bio-precipitation capacity and arsenic removal coupled with iron and manganese oxidation in filtration systems (**Table 1**) and recently, the use of acid/metal-tolerant sulfate reducing bacteria was applied for arsenic removal from an acid mine drainage (Serrano and Leiva, 2017).

As(III) microbial oxidation can also be coupled to commonly used adsorption removal technology, without any chemicals addition nor toxic by-products (Bahar et al., 2013). The As(III)-oxidation potentialities of several As(III)-oxidizing microorganisms, such as *Aliihoeflea* sp. 2WW, *Thiomonas arsenivorans* strain b6, *Ensifer adhaerens*, *Rhodococcus equi* and other As(III)-oxidizing mixed bacterial populations as planktonic cells or associated with biofilms were successfully tested in lab-scale experiments for treating contaminated water (**Table 1**). Moreover, the anoxic As(III) microbial oxidation coupled with chemolithotrophic denitrification was successfully employed in the treatment of arsenic in bioreactors (Sun et al., 2010). To date, only one case study of full-scale treatment of arsenic contaminated groundwater using biological As(III) oxidation has been documented in the scientific literature (Katsoyiannis et al., 2008). This multi-stage treatment method was based on the biological oxidation of NH_4_^+^ and Mn(II) for the simultaneous As(III) oxidation and subsequent As(V) removal by coagulation. However, As removal is strongly dependent on Fe(II) and Mn(II) concentrations since the process relies on the sorption of As on iron and manganese oxides produced by autochthonous Fe(II)- and Mn(II)-oxidizing bacteria.

Although several studies demonstrated the efficacy of arsenic removal from water by microorganisms, these approaches are yet to be fully exploited for arsenic remediation, and knowledge about the diversity and distribution of functional genes controlling arsenic transformation in such processes is still quite fragmentary (Andres and Bertin, 2016; Crognale et al., 2017). The industrial application of arsenic removal from water still requires further evaluation in real situation of additional aspects such as the influence on microbial As(III) oxidation of geometric and hydraulic parameters in column systems or the requirement for carbon supply to support fast reactions. Although recent batch experiment works are addressing the question of the effects of nutrient sources and temperature in acid mine drainage (Tardy et al., 2018), there is still a want of further genomic and metagenomic studies of arsenic-contaminated ecosystems addressing not only the metabolisms of metals, arsenic and sulfurs but the full-range of microbial metabolic capacities. Such studies will be necessary for understanding the complex trophic interaction network of microorganisms in those ecosystems and for designing optimized artificial microbial communities that could be exploited in large-scale arsenic remediation systems.

## Conclusions and Perspectives

At the interface between molecular biology and ecology, environmental genomic DNA sequencing techniques allow to reach, beyond the mere description of a simple organism, the characterization of complex microbial communities including organisms recalcitrant to isolation and culture. In association with global functional approaches – metatranscriptomics, metaproteomics, metabolomics including stable-isotope probing (Fischer et al., 2016; Musat et al., 2016; Vogt et al., 2016; Zuñiga et al., 2017) – these techniques help increasing our knowledge of the functioning of ecosystems. Additionally, the sequencing depth attained by these new technologies can give access to the less represented species of an ecosystem (the rare biosphere). Allowing fast and inexpensive massive characterization of microbial communities, they could also be an asset for the continuous monitoring of microbial communities involved in bioremediation processes to avoid changes that could compromise the efficiency of the treatment (Lovley, 2003; Stenuit et al., 2008; Techtmann and Hazen, 2016). In combination with the indispensable experimentations in the laboratory and in the field, these approaches require the development of efficient reproducible sampling and extraction methods as well as of robust and new computing solutions for storing, exchanging, and analyzing the huge amounts of data they produce. Indeed, power analysis and sample size requirements estimation for high-throughput sequencing data demand computations of much higher complexity than classical statistical analyses and must be fine-tuned to the type of problem that is being addressed (Pasolli et al., 2016; Li et al., 2017). It is moreover necessary that all published studies include complementary data (meta-data) which should be collected for every genome/metagenome to permit the proper exploitation of data (Satinsky et al., 2013) as defined by the Genomic Standard Consortium (Yilmaz et al., 2011).

The public access on sites like the EBI Metagenomics (Mitchell et al., 2017) to thousands of metagenomic samples combined with big data analysis, data mining algorithms and metabolic modeling constitutes an unprecedented opportunity to study and understand how the different components of an ecosystem may function together in relation with environmental biotic and abiotic factors, largely surpassing mere inventories of biological objects. A better understanding of the concerned organisms, of their spatial and temporal distribution, of the adaptive and evolutive processes at stake and of the metabolic interactions they develop should thus provide an integrated image of the microbial communities and metabolic functions involved in the microbiological processes underlying arsenic removal from water. Using *ad hoc* predictive models, such knowledge may be expected to permit the optimal utilization of microorganisms’ properties in biotechnological applications and bioremediation processes.

## Author Contributions

PB and FP organized the content of the entire manuscript and wrote the genomics section. SC and SR wrote the section on bioremediation.

## Conflict of Interest Statement

The authors declare that the research was conducted in the absence of any commercial or financial relationships that could be construed as a potential conflict of interest.
